# Analysis of baseline, average, and longitudinally measured blood pressure data using linear mixed models

**DOI:** 10.1186/1753-6561-8-S1-S80

**Published:** 2014-06-17

**Authors:** Ahmed Hossain, Joseph Beyene

**Affiliations:** 1 Department of Clinical Epidemiology and Biostatistics, McMaster University, 1280 Main Street West, Hamilton, Ontario L8S4K1, Canada

## Abstract

This article compares baseline, average, and longitudinal data analysis methods for identifying genetic variants in genome-wide association study using the Genetic Analysis Workshop 18 data. We apply methods that include (a) linear mixed models with baseline measures, (b) random intercept linear mixed models with mean measures outcome, and (c) random intercept linear mixed models with longitudinal measurements. In the linear mixed models, covariates are included as fixed effects, whereas relatedness among individuals is incorporated as the variance-covariance structure of the random effect for the individuals. The overall strategy of applying linear mixed models decorrelate the data is based on Aulchenko *et al*.'s GRAMMAR. By analyzing systolic and diastolic blood pressure, which are used separately as outcomes, we compare the 3 methods in identifying a known genetic variant that is associated with blood pressure from chromosome 3 and simulated phenotype data. We also analyze the real phenotype data to illustrate the methods. We conclude that the linear mixed model with longitudinal measurements of diastolic blood pressure is the most accurate at identifying the known single-nucleotide polymorphism among the methods, but linear mixed models with baseline measures perform best with systolic blood pressure as the outcome.

## Background

Hypertension is a major morbidity and mortality risk factor for stroke, myocardial infarction, heart failure, and end-stage renal disease [1]. It is a multifactorial disorder resulting from inheritance of several susceptibility genes, as well as multiple environmental determinants, including weight control, dietary intake, physical activity, and alcohol consumption [2]. To date, several variants have been identified by genome-wide association studies (GWAS) as being associated with blood pressure and hypertension [1,3,4]. Various statistical, data mining, and machine learning strategies have shown some promise for identifying genetic variants, but are not scalable to large-scale GWAS [5,6]. Linear mixed models (LMMs) are widely used in controlling for phenotypes and relatedness within GWAS [7]. In the application of LMMs for GWAS data the covariates are included as fixed effects, whereas kinship among individuals is incorporated as a variance-covariance structure of the random effect for the individuals. We followed Aulchenko *et al's *[8] residual approach, which is based on a 2-step strategy in the application of the LMM. The first step optimizes a reduced LMM with the genetic marker effect excluded. In the second step, the residual from the reduced LMM is fitted as the dependent variable to test each marker in a linear model.

We give an overview of 3 LMMs for the analysis of Genetic Analysis Workshop 18 (GAW18) data, paying attention to the power of selecting an associated single-nucleotide polymorphism (SNP) from chromosome 3 and simulated phenotype data. In particular, we apply 3 types of LMMs for statistical analysis of baseline measurements, mean measurements, and longitudinal data. We compare the LMMs through simulations and illustrate them using the real phenotype data.

## Methods

### Data and quality control

We use 3 models to analyze the GWAS data set from chromosome 3 of the GAW18: a Diabetes-GENES Project, which consists of whole genome sequence data in a pedigree-based sample, longitudinal phenotype data for hypertension and related traits, and selected covariates. In this GWAS data, 65,519 SNPs have been genotyped for chromosome 3. In the simulated phenotype data, 849 subjects were measured at 3 time points for age, medication use, smoking status, and blood pressure. As is standard practice, SNPs with minor allele frequency (MAF) <1% were excluded from data analysis. We also filtered out SNPs with low call rates (<90%) and deviation from Hardy-Weinberg equilibrium (*p *value ≤ 1*e*^−6^). The quality controls were implemented using the R package SNPassoc [9]. In addition, we excluded 4 individuals because more than half of their SNP values were missing. After filtering, a total of 27,313 SNPs and 845 samples met our quality-control criteria and were used for analysis. The family relationships among these individuals were copied from the pedigree on the real data.

### Statistical analysis to evaluate the effect of SNPs

We consider 3 LMMs to evaluate the effect of SNPs on systolic blood pressure (SBP) and diastolic blood pressure (DBP) separately.

### Model 1: GRAMMAR approach for baseline measures analysis

Aulchenko *et al*. [8] proposed a genome-wide rapid association using mixed model and regression (GRAMMAR) to assess significance of the effect of a polymorphism. The method first obtains residuals adjusted for family effects and then analyzes the association between these residuals and genetic polymorphisms using least-squares methods. The model is expressed as follows:

*Initial model*. The initial model is $y_{ij}=\beta_{0}+\underset{k}{\sum }\beta_{k}x_{ijk}+G+e_{ij}$, where *y_ij _*is the value of phenotype corresponding to the *j*^th ^individual in the *i*^th ^pedigree, *x_ijk _*is the value of the *k*^th ^covariate or fixed effect, $\beta_{k}$ is an estimate of the *k*^th ^fixed effect or covariate, and *e_ij _*is the vector of residual effects. *G *is the random polygenic effect that follows a multivariate normal distribution with mean 0 and variance $\text{Φ}\sigma^{2}G,$ where *Φ *is the relationship matrix (kinship matrix) and $\sigma^{2}G$ the additive genetic variance as a result of polygenes. The vector of estimated residuals is given by $y^{*}=y-\left({\hat{\beta }}_{0}+\underset{k}{\sum }{\hat{\beta }}_{k}x_{ijk}+\hat{G}\right)=\hat{e}$.

*SNP model*. The residuals are used as the dependent trait in a simple linear regression for each SNP, $\hat{e}=\alpha^{*}+\gamma_{1}SNP_{1}+\varepsilon$, where $\gamma_{1}$ is the coefficient of the *l*^th ^SNP from the model 1 scenario. The method adjusts for familial relationship and is computationally fast, but the model only considers the time 1 information from the GAW18 data. The first stage model is implemented using the polygenic() function of the R package GenABEL, and the kinship matrix is estimated using the R package kinship2. Next, the lm() function is used for fitting the linear model with residuals obtained from the first-stage model.

### Model 2: Two-stage LMM for mean measured outcome analysis

We considered the measurement of the mean across the 3 time points as the outcome and followed the 2-stage approach. The model formula for the first stage is ${\bar{y}}_{ij}=\beta_{0}+\underset{k}{\sum }\beta_{k}x_{ijk}+G+e_{ij}$, where ${\bar{y}}_{ij}$ denotes the mean phenotype across the time points for the *j*^th ^individual in the *i*^th ^pedigree. *β *is the coefficient for unknown fixed effects representing nongenetic effects (mean age across time points, sex, smoking status at time 1, and medication use at time 1), and *G *is the random polygenic effect that follows a multivariate normal distribution with mean 0 and variance $2K\sigma_{G}^{2},$ where *K *is the kinship matrix with elements $k_{ij}\left(j=1,2,\ldots ,n_{i}\right)$ calculated from pedigrees, and $\sigma_{G}^{2}$ is an unknown genetic variance; *e *is a vector of random residual effects that are normally distributed with zero mean and variance-covariance $R=I\sigma_{e}^{2},$ where I is the identity matrix and $\sigma_{e}^{2}$ is the unknown residual variance.

In the second stage we consider the residuals as the outcome and fit the linear model, $\hat{e}=\beta^{*}+\gamma_{2}SNP_{1}+\varepsilon ,$ where $\gamma_{2}$ is the coefficient of the *l*^th ^SNP from the model 2 scenario. We implemented the model using the R package kinship (R 2.10.1). The lmekin() function is used to obtain the residuals from the first-stage model.

### Model 3: Two-stage LMM for longitudinal analysis

We also evaluate a 2-stage LMM that takes longitudinal measurements into account. We consider 2 models, without and with time effects, in the application of the longitudinal analysis. In the first stage, we fit a random intercept LMM as follows:

*(A)$y_{ijt}=\beta_{0}+\underset{k}{\sum }\beta_{k}x_{ijtk}+G+e_{ijt}$*(1)

Next, we extend the first-stage model allowing time points:

*(B)$y_{ijt}=\beta_{0}+\underset{k}{\sum }\beta_{k}x_{ijtk}+vz_{t}+G+e_{ijt}$*(2)

where *y_ijt _*denotes the phenotype (SBP or DBP) for the *j*^th ^individual in the *i*^th ^pedigree at time t. *x_ijt _*is the k fixed effect time-dependent covariate, *v *is the slope coefficient for the time points *z_t_*; t = 1, 2, 3, and G is the random polygenic effect as in model 2; *e *is a vector of random residual effects that are normally distributed with zero mean and covariance $R_{0}=I\sigma_{e_{0}}^{2}$, and $\sigma_{e_{0}}^{2}$ is the unknown residual variance. Then we consider the mean of the residuals across the time points as the outcome and fit the model $\bar{e}=\beta^{**}+\gamma_{3}SNP_{l}+\varepsilon$ where $\bar{e}$ is the vector of mean residuals across the time points and $\gamma_{3}$ is the coefficient of the *l*^th ^SNP from the model 3 scenario. We applied the model using the R packages pedigreemm and kinship.

## Results

### Simulated data analysis

We investigated the performance of the 3 LMMs for selecting a known associated SNP from simulation studies. The 3 models are employed after adjusting for covariates and pedigree information, and the *p *values for each SNP are used to rank the SNPs.

The simulated phenotype data in GAW18 has 10 known SNPs from chromosome 3 that are associated with blood pressure. Among these SNPs, 2 have MAF >0.05. These 2 variants are rs6442089 (gene symbol: *MAP4*, position: 47956424, and MAF: 0.367) and rs1131356 (gene symbol: *FLNB*, position: 58109162, and MAF: 0.488). We investigated the 3 LMMs in terms of selection performances of rs6442089. We selected rs6442089 because it is a well-known SNP from the gene *MAP4 *that affects blood pressure.

**Figure 1:**
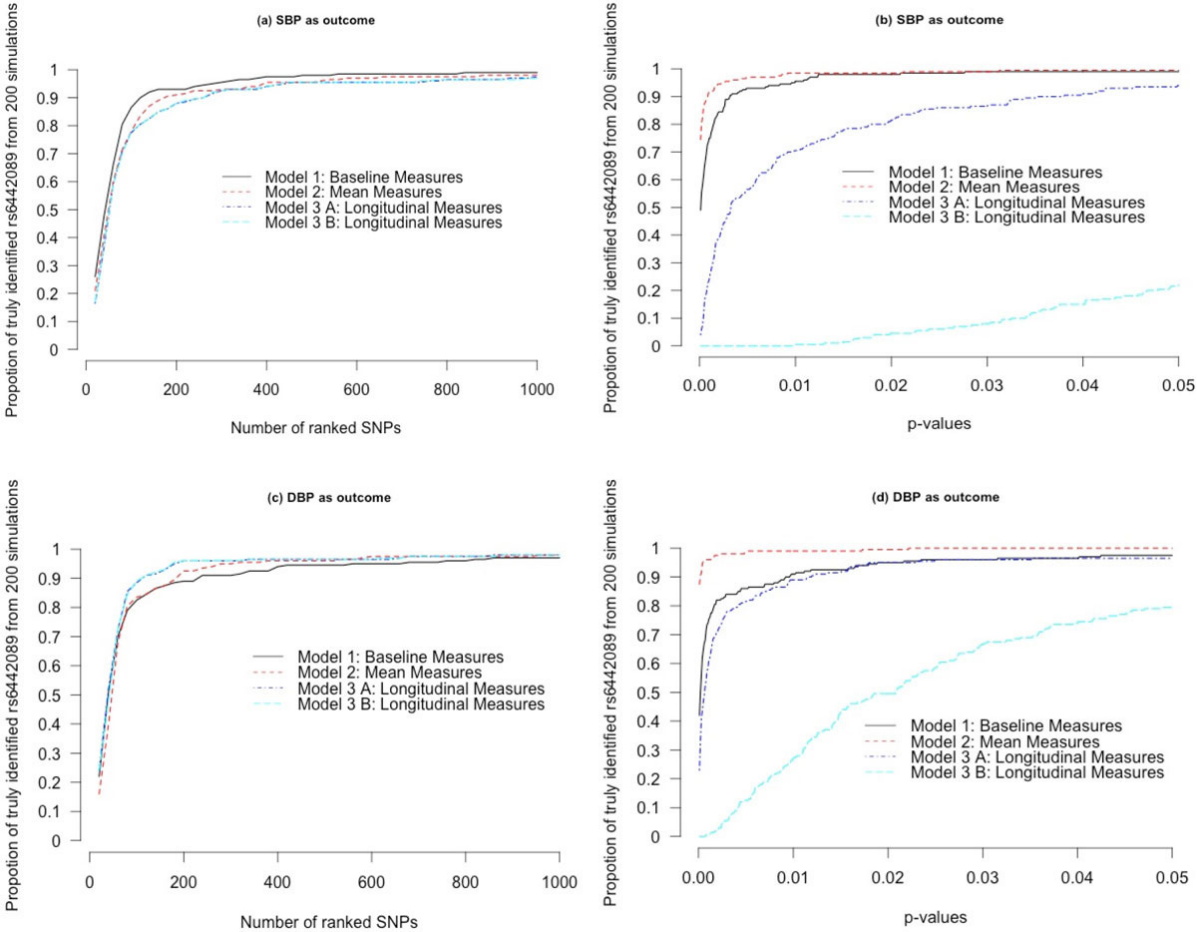
**Identifying a known significant SNP by three LMMs using the simulated GAW18 data**.

We denote a SNP to be significant either if its *p *value is smaller than a cutoff value or if it belongs to a target number of ranked SNPs. For example, if our target number of selected SNPs is 200, then a SNP will be called truly identified from a simulated phenotype data if its rank belongs to the top 200. Alternatively, if our target cutpoint for *p *value is 0.001, then a SNP will be called truly identified if the *p *value of the SNP is found to be less than 0.001. The proportion is estimated by counting how many times from the 200 simulations the SNP (rs6442089) was in the list of target SNPs or within the *p *value cutpoint. Figure 1 lists the proportions for the 3 methods. Figure 1A indicates that the GRAMMAR procedure with the baseline measures is more effective than any of the other methods in selecting the SNPs considering SBP as outcome. But we found that the GRAMMAR procedure was not effective with baseline DBP measures among the models (Figure 1C). As seen in Figures 1Band D, LMM with mean measures outcome has greater power to detect the genetic variant considering a cutpoint of *p *values. It appears from the figures that applying LMM to longitudinal DBP data provides better results in selecting the SNP compared to any of the other methods. We found similar results by both Model 3(A) and Model 3(B). That is, the results from Model 3(A) and Model 3(B) do not look qualitatively different from each other: In both cases, the performance of selecting the SNP is lower in SBP and higher in DBP.

### Application to real data

We employed the 3 LMMs to real phenotype SBP data after adjusting for covariates and we rank the SNPs using the *p *values for each SNP. We considered first 3 time points to avoid the missing values of the fourth time point, and we applied the 3 models to the same 845 individuals who were selected in the simulated data analysis. We report 5 top-ranked SNPs in Table 1. It can be seen that the *p *values from the LMM with longitudinal measurements are conservative compared to other methods. After investigation of the top 20 SNPs we found 3 SNPs in common across the models. The ranks for the known SNP, rs6442089, are 4376, 3105, and 758 by the LMMs with baseline, longitudinal (Model 3A), and mean measures outcome, respectively. Therefore, the real phenotype data suggest that the LMM with mean measurements performs best among the 3 methods for identifying the SNP rs6442089.

**Table 1:** Top 5 SNPs by the 3 LMMs considering the outcome SBP

Rank	Model 1 Baseline measures	Model 2 Average measures	Model 3A Longitudinal measures
1	rs2712464 (7.062e-06)	Rs9846213 (1.745e-05)	Rs9813958 (4.211e-04)
2	rs2953046 (9.053e-06)	Rs3911499 (2.500e-05)	Rs2662090 (1.586e-03)
3	rs1445065 (1.864e-05)	Rs17005789(2.813e-05)	Rs10511379 (1.651 e-03)
4	rs2867840 (2.708e-05)	Rs534185 (3.187e-05)	Rs12488556 (1.811e-03)
5	rs1386291 (3.357e-05)	Rs2161060 (4.696e-05)	Rs2366104 (1.810 e-03)

*p *Values are shown in parentheses.

## Discussion

In this article we applied 3 LMMs to the study of GAW18 in family data and in settings of relevance to baseline measures, mean measures, and longitudinally measured data. The statistical analysis of GWAS for GAW18 data using LMMs with longitudinal DBP measurements is capable of revealing the dynamic pattern of genetic control over chromosome 3 but did not perform competitively with other models for longitudinal SBP measurements. Exploratory/graphical analysis for the trajectories of SBP and DBP measurements also supported the conclusion that DBP had more subject-specific variability in slopes than SBP. However, the GRAMMAR approach with single-measure SBP data at baseline can be used on the development of SNP selection.

A general consideration applicable to all the methods discussed here concerns the issue of whether the outcome is linear or nonlinear. An alternative approach could be to relax the conditions imposed on linear models and explore the hidden structure by using a varying coefficient model [10]. Consequently, it will be interesting to apply another method assuming the effects of SNPs are smooth functions of time.

## Conclusion

We showed that a linear mixed modeling framework was most accurate at identifying known single-nucleotide polymorphism compared to other competing methods we considered in this manuscript for the analysis of longitudinal measurements of diastolic blood pressure. In contrast, baseline measures performed best with systolic blood pressure highlighting that, depending on the trajectory profile of the quantitative trait of interest, either just baseline values or serially measured values can be useful in genetic association studies.

## Competing interests

The authors declare that they have no competing interests.

## Authors' contributions

AH designed the overall study, performed all of the data analysis and drafted the manuscript. JB assisted in conceiving the idea, helped in drafting the manuscript and provided overall supervision. Both authors read and approved the final manuscript.
